# Colorectal endometriosis: Diagnosis, surgical strategies and post-operative complications

**DOI:** 10.3389/fsurg.2022.978326

**Published:** 2022-10-04

**Authors:** Saeed Alborzi, Horace Roman, Elham Askary, Tahereh Poordast, Mahboobeh Hamedi Shahraki, Soroush Alborzi, Alimohammad Keshtvarz Hesam Abadi, Elnaz Hosseini Najar Kolaii

**Affiliations:** ^1^ Department of Obstetrics and Gynecology, School of Medicine, Laparoscopy Research Center, Shiraz University of Medical Sciences, Shiraz, Iran; ^2^ Department of Gynecology and Obstetrics, Clinique Gynécologique et Obstétrical, Rouen University Hospital, Rouen, France; ^3^ Department of Obstetrics and Gynecology, School of Medicine, Infertility Research Center, Shiraz University of Medical Sciences, Shiraz, Iran; ^4^ Department of Obstetrics and Gynecology, Shiraz University of Medical Sciences, Shiraz, Iran; ^5^ Cardiologist, Student Research Center, School of Medicine, Shiraz University of Medical Sciences, Shiraz, Iran; ^6^ Master of Biostatistics in Clinical Research Development Center of Nemazee Hospital, Department of Statistics, Shiraz University of Medical Sciences, Shiraz, Iran

**Keywords:** endometriosis, rectal endometriosis, Clavien-Dindo classification, infertility, rectovaginal fistula, painful menstruation

## Abstract

**Objective:**

The present work aimed to investigate the feasibility, complications, recurrence rate, and infertility outcomes of the radical and conservative surgical methods for colorectal endometriosis in short- and long-term follow-ups.

**Methods:**

In this prospective study, the patients with confirmed diagnosis of colorectal DIE were included from March 2015 to March 2021, who were referred to an Endometriosis Surgery Center affiliated with Shiraz University of Medical Sciences (SUMS). Information on demographics, surgical approaches, intra-operative, and post-operative findings as well as complications were collected and compared. Six- and 12-month interviews were conducted to evaluate the functional outcomes of all the procedures.

**Results:**

Out of 3,111 patients who underwent endometriosis surgery, 837 (28.19%) with the average age of 34.2 ± 5.9 years and average ASRM score of 102.1 ± 36.8 had rectosigmoid endometriosis. Laparoscopic rectal shaving was performed in 263(30.0%) patients while 326 (37.2%) underwent segmental bowel resection, and 248 (28.30%) were treated with disc excision. Prophylactic ileostomy was performed in six (0.68%) patients and peritonitis was reported in four (0.45%). Five (0.58%) subjects developed rectovaginal fistula and one (0.11%) was diagnosed with bladder atonia. The recurrence rate was 3.8%, 1.2%, and 0.3% in rectal shaving, disc, and segmental bowel resection techniques, respectively. Dysmenorrhea, dyspareunia, and dyschezia were improved after surgery by 7.3, 9.4, and 12.5 times, respectively. We observed 25.2% of total pregnancy following the operation, the majority of which occurred in the first year after the surgery.

**Conclusion:**

There were very few short-term or long-term complications in the three different techniques when the choice was correct.

## Introduction

The prevalence of endometriosis is about 10% in the reproductive age. The prevalence of colorectal endometriosis was reported to be 8%–12% with 90% of the lesions seen in the recto-sigmoid region (1–5). According to ASRM (American Society for Reproductive Medicine) classification, rectal endometriosis often presents with severe forms of endometriosis. Symptoms like dysmenorrhea, dyschezia, dyspareunia, constipation, and tenesmus are associated with colorectal endometriosis, all of which can significantly affect a person's quality of life (6).

In patients with any symptoms of intestinal obstruction, or those who do not respond to medical therapy, surgical treatment is carried out through laparoscopic or robotic methods (7–9). Currently, the three suggested surgical treatment methods for rectal endometriosis include the shaving technique, disc excision (DR), which is considered a conservative method, segmental bowel resection (SR), and re-anastomosis, which is an intense method. Based on recent studies, the existing methods are different in terms of short- and long-term complications, the risk of recurrence, and functional outcomes. Additionally, there is still no clear agreement among gynecologist surgeons on the optimal patient management for these lesions (10–13).

Today, radiological methods have made it possible to determine the nodule size, its location and multifocality, and the percentage of lumen stenosis prior to surgery. Based on this information, we are able to determine which patients will benefit from radical surgery (14, 15).

Currently, there is scarce research on the feasibility, complications, recurrence rate, and infertility outcomes of the three available surgical methods in short- and long-term follow-ups. The present study included a large number of colorectal endometriosis surgery cases so that all these three surgical procedures could be compared in terms of all the short- and long-term outcomes.

## Materials and methods

### Study population

This was a prospective study performed on the patients referred to an Endometriosis Surgery Center affiliated with Shiraz University of Medical Sciences (SUMS) with a suspicious history of endometriosis. The patients diagnosed with colorectal endometriosis using imaging techniques were recruited from March 2015 to March 2021. SUMS Institutional Review informed consent was taken from all the subjects before participation.

The inclusion criteria were (1) the age range of 18 to 50 years old and one of the followings: (2) endometriosis-related pain without response to medication, (3) involvement of tubes in case of infertility, (4) complete family planning according to decision making by couples, (5) conditions where using hormonal drugs is not possible, (6) unexplained infertility with AMH 1–2 ng/ml, (7) unilateral OMA with AMH >2.5, (8) more than two unsuccessful IVF attempts, (9) the need for pathology samples, and (10) patient's unwillingness to receive medical treatment. All the surgeries, as well as all IVF procedures, were performed by the first author. The exclusion criteria were (1) all the patients with cardiovascular diseases, (2) insulin-dependent diabetes mellitus, (3) BMI (Body Mass Index) >35 kg/m^2^, and (4) a history of pelvic organ malignancy. The endometriosis-related pain symptoms, such as dysmenorrhea, dyspareunia, dyschezia, mid cyclic or non-cyclic pain, constipation, as well as other GI troubles (Gastro-Intestinal) were recorded before the operation and every 6 months afterwards based on VAS score (Visual Analogue Scale) (16).

The recorded demographic information and medical history of the patients included age, BMI, parity, history of previous operation and endometriosis surgeries, pre- and post-operation pain symptoms related to endometriosis, intra- and post-operation complications according to Cliven–Dindo classification, stage of endometriosis disease according to ASRM classification (17, 18), histo-pathologic report, recurrence rate of disease, pregnancy rate, pregnancy outcome, and the method of pregnancy. These data were compared between the three different surgical procedures performed on colorectal endometriosis lesion, namely shaving, disc resection and segmental bowel resection, and re-anastomosis.

The patients were followed for 6 months after the surgery and then annually. We used histopathology specimens as the gold standard of diagnosis for rectal endometriosis.

All the surgeries were performed laparoscopically and according to the guidelines with nerve and vascular sparing techniques. In our work, superficial DIE lesions of bowel were removed by shaving, deep lesions between 1 cm–3 cm were operated by disc excision, and multiple and large (>3 cm) lesions with >50% involvement of bowel loop circumference were removed by segmental resection (2, 16, 18). None of the surgeries were converted to laparotomy except for two cases of internal iliac injury.

### Statistical analysis

All the data were entered into a database and further analyzed using the statistical package for social sciences (SPSS Inc. version 24.0). Data are presented as mean ± SD or proportions. Parametric variables with normal distribution were compared using one-way analysis of variance (ANOVA) with Tukey, as the *post hoc* test. The non-parametric variables were compared using Kruskal-Wallis test. The groups were compared with t test (for continued variables) and chi-square for the categoric variables. A two-sided *P*-value of less than 0.05 was considered to be statistically significant.

## Results

Out of 3,111 patients referred with endometriosis to our center in the study period, 837 underwent rectal endometriosis surgery. Out of this population, 263 (30.0%) underwent shaving, 248 (28.3%) DR, and 326 (37.2%) SR. Among all the patients, we had only six cases of cecal involvement. Two cases of small bowel involvement were reported, who previously had laparotomy endometriosis pelvic surgery.

The patients’ demographics, medical history and endometriosis-related pains for each group are summarized in Table 1.

**Table 1:** Demographic data of all three groups of surgeries.

	Shaving *N* = 263 (29.9%)	DR *N* = 248 (28.27%)	SR *N* = 326 (37.1%)	*P* Value
Age (year) (mean, 95% CI)	34.13 (33.20–35.07)	34.80 (33.61–35.99)	35.37 (34.67–36.00)	0.15
Previous endometriosis surgery (%)	25%	35%	39%	
>1 Previous endometriosis surgery (%)	2.9%	5%	2.1%	0.052
Interval between surgery (month)	24–38	36–38	38	0.78
Score of disease according to ASRM (Mean)	103.51 (95% CI 98.22–108.81)	111.40 (95% CI 104.5–118.30)	111.62 (95%CI 107.46–115.78)	0.25
Blood group O+	32.1%	37.8%	36.7%	
Blood group A+	30.7%	20.8%	28.3%	0.47
Pre operation constipation	10.7%	10.7%	14.8%	0.042
Post operation constipation	5%	8.9%	13.1%	
Pre operation bloating	1.2%	3.6%	1.7%	0.073
Pre operation diarrhea	0%	0%	1.1%	1.000
Pre operation Non cyclic pain	17.5%	7.5%	14.7%	0.066
Pre operation Mid cyclic pain	4.8%	0.6%	2.3%	0.060

**Figure 1:**
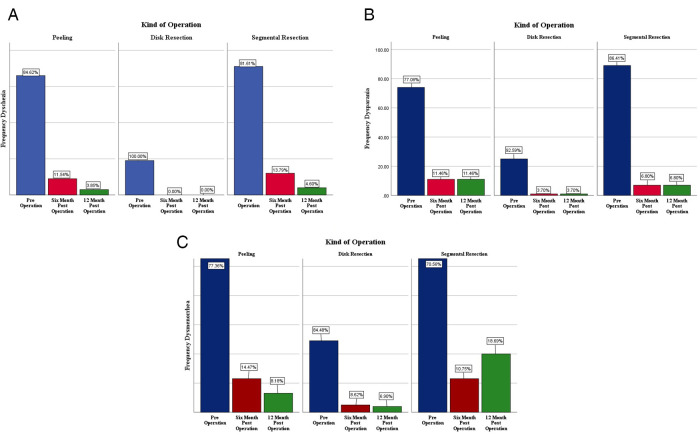
Comparison of dyschezia (**A**), dyspareunia (**B**) and dysmenorrhea (**C**), before surgery, six and 12 months after surgery based on the type of colorectal endometriosis surgeries.

The most common symptoms reported by the patients were dysmenorrhea (94.1%). All their initial symptoms and percentage of recovery after the surgery, based on the type of operation, are listed Figure 1. Twelve months following the operation, the intensity of dysmenorrhea decreased from 77.46% down to 11.34%, the dyspareunia declined from 85.99% to 7.25, and dyschezia decreased from 88.8% to 2.81% in all the patients, compared to the pre-operative period.

The frequency of pelvic lesions and surgeries, based on the method of operation and histopathologic findings are shown in Tables 2, 3. Rectal endometriosis lesions were the most prevalent in the presence of the uterine sacral ligaments DIE as well as cases with obliteration of the posterior cul-de-sac.

**Table 2:** Percentage of endometriosis involvement in different parts of the pelvis simultaneously with colorectal endometriosis according to histopathologic findings.

Operation finding	Percentage
Right endometrioma	61.2
Left endometrioma	58.3
Right ovarian fossa	42.4
Left ovarian fossa	40.2
Right uterosacral ligament	83.3
Left uterosacral ligament	81.2
Anterior cul desac	3.1
Posterior cul desac	97
Right hydrosalpinx	35.9
Left hydrosalpinx	40.5
Retrocervical	60.6
Vagina	3.9
Bladder	1.7
Ureters	17.3
Cecal involvement	0.68
Small bowel	0.22
External iliac artery	0.11
Nerve root	0.45

**Table 3:** Frequency of endometriosis surgery in different parts of the pelvis, the Cliven–Dindo Type II and III surgical complication and percentage of pregnancy after surgery based on different types of colorectal endometriosis surgeries.

Type of operation	Shaving	DR	SR	*P* value
**Frequency of endometriosis surgery in different parts of the pelvis based on different surgical methods**				
Hysterectomy; (%)	16.1	18.2	3.5	<0.0001
Right salpingectomy; (%)	41.3	50.6	47.7	>0.05
Left salpingectomy; (%)	51.7	56.5	53.5	>0.05
Right Oophorectomy; (%)	14.8	16.9	3.9	0.003
Left oophorectomy; (%)	17.8	18.8	2.7	0.014
Right ureterolysis; (%)	11.3	9.8	14.1	>0.05
Left ureterolysis; (%)	10.0	9.8	15.6	>0.05
Ureteral reanastomosis; (%)	0	0.7	2.0	
**Frequency of the Cliven–Dindo Type II and III surgical complication**				
Operation time (hour); mean (CI 95%)	1.95 (1.81–2.08)	2.36 (2.17–2.50)	3.68 (3.51–3.84)	<0.0001
Hospitalization (day); mean (CI 95%)	4.39 (4.14–4.64)	5.97 (5.71–6.24)	7.39 (7.16–7.62)	<0.0001
Hemoglobin drop (mg/dl); mean (CI 95%)	1.91 (1.75–2.07)	2.17 (1.97–2.37)	2.59 (2.44–2.74)	0.001
Fever (%)	17.2	15.6	60.9	0.018
Operation time (hour); mean (CI 95%)	1.95 (1.81–2.08)	2.36 (2.17–2.50)	3.68 (3.51–3.84)	<0.0001
Transfusion; (%)	21.9	9.6	65.2	<0.0001
External iliac artery injury (%)			0.92 (*n* = 3)	N/A
DVT (%)			0.30 (*n* = 1)	
Peritonitis (%)	0.76 (*n* = 2)	0.40 (*n* = 1)	0.30 (*n* = 1)	0.789
Abscess (%)			0.30 (*n* = 1)	
RVF (%)		0.40 (*n* = 1)	1.22 (*n* = 4)	
Early bladder atony <3 months (%)			0.92 (*n* = 3)	
Late bladder atony <3 months (%)			0.30 (*n* = 1)	
**Percentage of pregnancy after surgery**				
Infertile patients; (%)	28.13	18.14	31.28	0.44
Failed IVF before surgery; (%)	29.6	17.0	51.1	0.61
Time to pregnant after surgery (month); Mean (CI 95%)	14 (5.02–22.97)	11.42 (7.0–23.63)	13.3 (5.97–20.62)	0.974
Clinical pregnancy rate; *N* (%)	24/76 (31.57)	9/45 (20)	13/102 (12.74)	0.081
Abortion rate; *N* (%)	2/76 (2.63)	2/45 (4.44)	2/102 (1.96)	0.344
Ongoing Pregnancy rate; *N* (%)	22/76 (28.9)	7/45 (15.55)	11/102 (10.7)	0.344

DVT, deep vein thrombosis; RVF, recto-vaginal fistula; *N*, number of patients.

All the patients were in stage 3 and 4 of the disease according to ASRM classification (3.97 ± 0.18).

In the segmental resection group, rectal involvement was deeper compared to that in the other groups (*P* = 0.000).

Table 3 also represents the post-operation complications according to Cliven–Dindo classification.

Fever and the need for blood transfusion were 17.2, 15.6, 60.9%, and 21.9, 9.6, 65.2% in the peeling, DR, and SR groups, respectively (*P* < 0.01). Certain complications, such as peritonitis and rectovaginal fistula, were 0.76, 0.40, 0.40% and 0.0, 0.4, 1.22% in the peeling, DR, and SR groups. Other complications, like abscess, external iliac artery injury, early and late bladder atony, and DVT, were seen in 2.7% of the patients and only in the segmental group.

Type II of the Cliven–Dindo operation-associated complications was significantly higher in the SR group (*P* = 0.037).

In case of multifocal rectal involvement, two separated nodules were reported in 50 cases (5.97%), three-point involvement in 12 cases (1.43%), and four-point in two cases, all in the SR group (0.23%). We had no reports of malignancy in our study.

Prophylactic iliostomy was performed in only five cases in the SR group, whose rectal lesion was very low (below 8 cm from the anal verge) or whose hysterectomy or vaginal lesion was removed at the same time as the rectal lesion excision.

The recurrence rate, based on the follow-up ultrasound and symptoms recurrence, was 3.8% (*n* = 10) in the peeling method, 1.2% (*n* = 3) in the DR, and 0.3% (*n* = 1) in the SR methods; this rate was significantly lower in the SR method (*P* = 0.008).

Herein, 276 out of 837 subjects were categorized as an infertile group, out of whom 17 were over the age of 42, 10 had undergone bilateral salpingo-ophorectomy, and two had severe male factor infertility. Twenty-four patients were lost to the follow-up. In total, out of 223 patients, 24 (24/76) in the peeling group, nine (9/45) in the disc group, and 13 (13/102) in the segmental group were infertile. Table 3 depicts the clinical pregnancy, abortion, and ongoing pregnancy rate and other information about pregnancy outcomes. The majority of the spontaneous pregnancies were seen in the peeling group (*n* = 21, *P* = 0.059); 72% of them became pregnant in the first year after the surgery and the rest got pregnant up to 60 months afterwards. There were no significant differences between the groups in terms of IVF failure before the surgery (*P* = 0.61). Comparing the pregnancy rate before and after the surgery, our results showed a significant improvement only in the peeling group (*P* = 0.005).

## Discussion

Given the fact that the proper surgical treatment for rectal lesions still remains controversial in different patients, having a large database with a large sample size will undoubtedly help surgeons to make a better decision about each patient individually (19–24). In this study with a large sample size, we examined all the short- and long-term outcomes as well as fertility results and draw comparisons between the two radical and conservative surgical methods. We showed that by choosing the right patient for each technique, all these methods are feasible and could be accompanied by excellent and acceptable outcomes.

Regarding the weakness of our study, we could mention the lack of comparison of hormone therapy after the surgery in the three groups and its effect on disease recurrence in addition to the lack of comparison of the lifestyle and behavior of the patients in the management of complications after the surgery. There are some other weaknesses, including loss of patients in the follow-up, which impacts the recurrences and pregnancy rate, unbalanced and discontinuous hormonal therapy intake, unbalanced length of the follow-up, the lack of randomization between the three techniques, leading to incomparable three subpopulations.

This study had several strengths, including having more colorectal endometriosis surgeries per year compared to most similar studies (146/year). Additionally, all the ultrasound imagings, surgeries, IVF procedures, examinations of patients in terms of disease recurrence, and surgical treatments of complications were often done by the first author himself. In this study, all the short- and long-term complications of the surgery were collected and reported based on the three different surgical methods. Despite the large number of cases and not having performed prophylactic ileostomy, we observed fewer surgical complications and lower recurrence rate compared to similar studies in this field although the follow-up period in our study was longer (up to 6 years).

In the current work, the history of previous endometriosis surgeries was higher in the SR group (*P* = 0.06). This may indicate incomplete initial surgery and the use of conservative methods in young patients, which may lead to recurrence of lesions and re-operation during the reproductive age. Abrão et al. recommended the use of further radical methods in younger patients and reserved the conservative methods for pre-menopausal women (25). In a study by Afros et al., patients with a nodule >3 cm had an RR of 2.5 (95% CI, 1.66–3.99) of the requiring bowel resection so that the recurrence of lesions would be reduced (26).

All the endometriosis-related pain symptoms, showed a significant reduction after the surgery in the present study. The symptoms did not show any significant increase or decrease when the patients were followed-up for at least 12 months compared to the first 6 months after the surgery. Similarly, Roman et al. reported a significant reduction in endometriosis-related pain symptoms immediately after the surgery (27). Turco et al. showed that after segmental resection of colorectal endometriosis lesions, most of the pain symptoms related to endometriosis, on top of the quality of life of patients according to the EHP-30 questionnaire, had a considerable improvement that was more evident in patients with multinodular rectal lesions (28). Thus, it can be concluded that with complete surgery, patients' pain will be significantly reduced and their quality of life will increase and remain constant in long-term follow-ups (29, 30).

In the present work, despite the higher number of Cliven–Dindo II complications in the patients with radical surgery, type IIIa and IIIb complications were less common compared to those in other studies (18).

Our results (Table 3) were in accordance with those reported by Benifallah et al. systematic review and meta-analysis, where the mean operation times of 203 and 258.7 min for DR and SR and the mean hospital stay of 5 and 7 days were reported. Although they observed better results for disc than segmental resection in the terms of operation time and hospitalization days, the differences between the two groups were not statistically significant (*P* = 0.99, *I*^2^ = 71%) (31). According to previous papers, since rectal shaving is less invasive, it is associated with a lower risk of immediate pre- and post-operative complications compared to the other two methods (8, 12, 32). However, this should not be considered as a reason to suggest rectal shaving for all patients regardless of other consequences, such as the need for more incidence of reoperation and recurrence rate of disease.

Leakage and peritonitis were reported in only four cases, with 0.7% in the peeling group, 0.4% in the DR, and 0.3% in the SR group (*P* = 0.78). In a systematic review by Meuleman et al., who included 49 articles with 2,036 patients, the chance of anastomosis leakage was reported to be about 1.3% (14). However, in line with our results, in the systematic review by Bendifallah et al., the chance of leakage followed by peritonitis was 0.2 in peeling, 1.0% in disc, and 1.9% in segmental bowel resection, with no differences between the three groups (*P* = 0.32) (31). In a multi-center study on 4,721 patients, the chance of early anastomosis leakage was 0.4%, emphasizing that the prevalence of radical surgeries in that study was very low (3.8% underwent SR and 58% peeling) (33). Nisolle et al. studied 177 cases of segmental and peeling patients and compared them in terms of Cliven–Dindo IIIb. Only one case of anastomosis leakage was reported in the segmental group (2%), which was caused by tension on the anastomosis. It seems as if by maintaining vascularity and pelvic nerves during surgery and proper mobilization of the anastomosis and checking the integrity of the anastomosis before the end of the surgery, certain complications, such as anastomotic leakage, can be prevented (34). In order to investigate vascular damage and subsequent ischemia that leads to fistula formation at the site of rectal or ureteral anastomosis, Ianieri et al., in a systematic review based on the results of eight studies, concluded that indocyanin during surgery is a useful tool for evaluating suitable blood supply of intestinal anastomosis. In their study, Hernández et al. qualitatively confirmed the value of intraoperative indocyanine use to predict fistula formation in full-thickness intestinal resection and re-anastomosis due to insufficient vascularization of the anastomosis site, with a sensitivity and specificity of 100%. However, the obtained results in this field are still inconsistent and there is a need for larger studies with a larger sample size (35, 36).

All our five RVF cases, four of whom required surgical intervention, had a simultaneous colpotomy incision.

The reason behind the occurrence of the RVF is the simultaneous colpotomy incisions at the time of rectal lesions removal. Our RVF rate (0.4%) was obviously lower than that reported in similar articles despite not having performed prophylaxis iliostomy. The risk of RVF following colorectal endometriosis surgery in a systematic review by Balla et al. was reported to be about 2.4% (37). In a recently published systematic review, the chance of an RVF in segmental resection of bowel was reported to be about 2.7%, which is associated with low rectal lesions and simultaneous clopotomy incisions (32). In a study by Bendifallah et al., there was no difference in the risk of RVF between disc and segmental resection (*P* = 0.76, *I*^2^ = 0) (31). Meanwhile, in our study, although not statistically significant, most cases of fistula were reported in the segmental group.

To prevent RVF in patients with colorectal endometriosis, it is necessary to pay attention to the following points: (1) preservation of the serosal surface of pelvic organs; the lower rectal lesions, which lack the serosal surface and whose resection requires extensive dissection of the pararectal space, are associated with a higher chance of RVF; (2) patients with positive air leakage test who require more manipulation and suturing during operation; (3) avoiding simultaneous surgery on rectum and vagina; (4) surgeon's experience (38).

About the other complications in our study, in the SR group, we found one case of late bladder atonia (0.1%) which improved within 3 months of self-catheterization.

In a recently published systematic review, the rate of late bladder atony has been reported to be 6.6% in SR, 4.1% in DR, and 0.4% in peeling groups (39). The majority of the reports in other studies on the prevalence of urological morbidities was shown to be low and unknown, which is due to the short follow-up time (40). As we know, about 72% of these complications will be resolved during the follow-up period (13). Only in a few case series, post-operative urinary problems were reported to be between 0% and 5% among rectal endometriosis surgery (22, 41, 42). In the research by Hernandez et al., over a period of 38 to 48 months, comparing 76 patients in the SR group, 20 in the DR group, and 47 in the shaving group, only 5.2% showed urinary incontinency and 2.6% presented fecal incontinency only in the SR group (43).

With regards to the LARS symptoms and examining certain symptoms, including pre- and post-operation constipation, post-operation bloating and diarrhea, and incomplete emptying of the rectum or fecal incontinency, in our study, only post-operative constipation did not significantly improve in the segmental group (from 14.8% into 13.1%) despite the improvement in the peeling (10.7% into 5.0%) and disc resection groups (10.7% into 8.9%) (*P* = 0.042).

LARS symptoms are more prevalent in case of removing very low rectal lesions (<9 centimeters distance from anal verge). As reported by Bafort et al., the length of removed rectosigmoid was not found to be related to post-operative LARS symptoms, and the symptoms of the intestine and bladder after the surgery were not significantly different between disc and segmental resection groups (44). Roman et al. suggested that conservative surgery, such as disc resection with trans anal stapler, can reduce the length of rectum removed, leading to less damage to the pelvic vasculature and nerves. As a result, post-operative LARS symptoms can be reduced as well (45). Despite accepting Roman's theory, which was conducive to removing rectal lesions over the recent years, our study concluded that segmental resection has no more complications than other conservative methods, which should not be neglected by an experienced surgeon.

In our study, the recurrence rate was significantly lower in the SR method (*P* = 0.008). Hernandez et al. reported a 12.7% recurrence rate in the peeling group, 5% in the DR, and 1.3% in the SR group (*P* = 0.052). The chance of recurrence was the highest in the peeling group, which is in agreement with our study (43). In a systematic review by Bendifallah et al., similar to ours, the risk of recurrence in the segmental group was lower compared with rectal shaving (*I*^2^ = 0%, *P* = 0.001); nonetheless, the recurrence rate did not differ between disc (*n* = 106) and segmental (*n* = 229) resection techniques (*I*^2^ = 0%, *P* = 0.11). However, these results were obtained only from three out of the 41 articles included in their study. In this paper, the recurrence time was reported between 12 and 94 months after the operation (39). In agreement with our observations, in a randomized clinical trial by Roman et al., although the chance of recurrence in the DR was reported to be slightly higher than that of the SR, the quality of life was the same in both groups (27). In the systematic review by Giampaolino et al., the temporarily suspension of both ovaries to the abdominal wall after surgery of stage 3 and 4 of endometriosis was shown to prevent moderate and severe adhesions after the surgery. Nevertheless, in the case of colorectal endometriosis surgeries, there is insufficient information on colorectal endometriosis surgeries for the necessity of placing anti-adhesion devices at the end of the operation or temporary suspension of both ovaries to the anterior abdominal wall in order to reduce the adhesion formation and complications of re-operation in these patients in case of recurrence of the disease (46). Multifocal lesions are sometimes far from the primary lesion without any adhesion to the first lesion and can be missed easily without intestinal palpation during surgery. Such lesions might require the en-block removal of a big part of intestine (47, 48). Bowel palpation for finding multiple bowel lesions during laparoscopy is a step that should not be neglected.

In conclusion, if there is a surgical indication for removal of the endometriosis lesions, complete and definitive resection of the lesions is recommended. All the removal procedures for colorectal endometriosis, if performed with the appropriate indication and in the appropriate individual, are associated with improved endometriosis-related symptoms, reduced recurrence rate, and very few complications.

In order to choose the best treatment method for rectal endometriosis lesions with a few surgical complications, further investigation is needed with the introduction of new surgical techniques as well as a larger sample size.

## Data Availability

The raw data supporting the conclusions of this article will be made available by the authors, without undue reservation.
